# Persistent Apical Akinesis Following Takotsubo Syndrome With Marked Troponin Elevation: A Case Report

**DOI:** 10.7759/cureus.117114

**Published:** 2026-09-28

**Authors:** Shreyas Mishra, Mrugesh B Patel, David Crowder, Sahil Sharma

**Affiliations:** 1 Cardiology, Patel Cardiovascular Consultants, Langhorne, USA; 2 Otolaryngology, Children's Hospital of Pittsburgh, University of Pittsburgh Medical Center, Pittsburgh, USA

**Keywords:** broken-heart syndrome, cardiac troponin, coronary artery angiography, echocardiogram, hiatal hernia, high-sensitivity cardiac troponin (hs-ctn), stress-induced cardiomyopathy, takotsubo syndrome, takotsubo syndrome vs. acute myocardial infarction

## Abstract

Takotsubo syndrome (TTS) is typically characterized by transient left ventricular (LV) systolic dysfunction with recovery occurring within days to weeks. Persistent regional wall motion abnormalities beyond the expected recovery period are uncommon. We report the case of a 77-year-old woman who presented with nausea, vomiting, epigastric discomfort, dyspnea, ST-segment depressions, and markedly elevated high-sensitivity troponin (9,848 ng/L). Coronary angiography demonstrated nonobstructive coronary artery disease, while echocardiography confirmed TTS with apical akinesis and an LV ejection fraction of 35%. A large hiatal hernia was also identified. Although the patient experienced complete symptomatic recovery and returned to baseline functional status, serial echocardiography demonstrated persistent apical akinesis through four months of follow-up. This case highlights an unusual recovery pattern in TTS and demonstrates that marked troponin elevation can occur despite nonobstructive coronary artery disease.

## Introduction

Takotsubo syndrome (TTS), also referred to as stress cardiomyopathy or broken heart syndrome, is characterized by acute, transient left ventricular (LV) systolic dysfunction. Although its pathophysiology remains incompletely understood, excessive catecholamine release, myocardial inflammation, and microvascular dysfunction are believed to contribute to the syndrome [1]. Patients typically present with chest pain, dyspnea, or syncope, often mimicking acute coronary syndrome (ACS). The diagnosis is commonly established using the International Takotsubo criteria [2], which incorporate transient regional wall motion abnormalities extending beyond a single epicardial coronary territory, the presence of an emotional or physical trigger, new electrocardiographic changes, moderate elevation in cardiac biomarkers, and exclusion of infectious myocarditis. Notably, these criteria do not require the absence of coronary artery disease. While troponin elevation occurs in TTS, levels are generally lower than in ACS [3], and LV function commonly recovers within days to weeks, with most patients demonstrating recovery by 12 weeks [4]. However, the acute phase can be complicated by hemodynamic or electrical instability, and increasing evidence suggests that long-term outcomes may parallel those of ACS [5].

Although lingering cardiac dysfunction and elevated troponin levels have been reported in TTS [6], persistent regional wall motion abnormalities extending beyond the typical recovery period have been described mainly in isolated case reports [7-10]. We present a case of a 77-year-old woman with TTS demonstrating persistent apical akinesis and reduced left ventricular ejection fraction (LVEF) more than four months after the acute event, accompanied by marked high-sensitivity troponin (hs-troponin) elevation (9,848 ng/L) and a large hiatal hernia.

## Case presentation

A 77-year-old woman with hypertension and hyperlipidemia presented to the emergency department with nausea, vomiting, and epigastric discomfort after driving for more than 10 hours on a hot day. She also reported chronic, mild left shoulder discomfort of unclear etiology. On arrival, she was hemodynamically stable with a blood pressure of 134/67 mm Hg, heart rate of 78 bpm, respiratory rate of 22 breaths/minute, and no fever. She had no prior cardiac history or testing.

CT excluded pulmonary embolism but revealed a large hiatal hernia with most of the stomach within the thoracic cavity, initially considered a cause of her symptoms. Despite this finding, persistent dyspnea, left shoulder pain, ST-segment depressions, and markedly elevated hs-troponin levels (9,848 ng/L) prompted urgent cardiac catheterization. This revealed a codominant coronary system with mild, nonobstructive coronary artery disease: an approximately 20% narrowing of the left anterior descending (LAD) artery just beyond the first diagonal branch with some haziness, with the remaining vessels smooth and tortuous but without significant disease. Flow was preserved in all vessels, and LV end-diastolic pressure was severely elevated. Echocardiography in the recovery unit confirmed TTS with apical akinesis and an LVEF of 35% on Day 1, calculated using Simpson's biplane method (Figures 1A, 1B).

**Figure 1:**
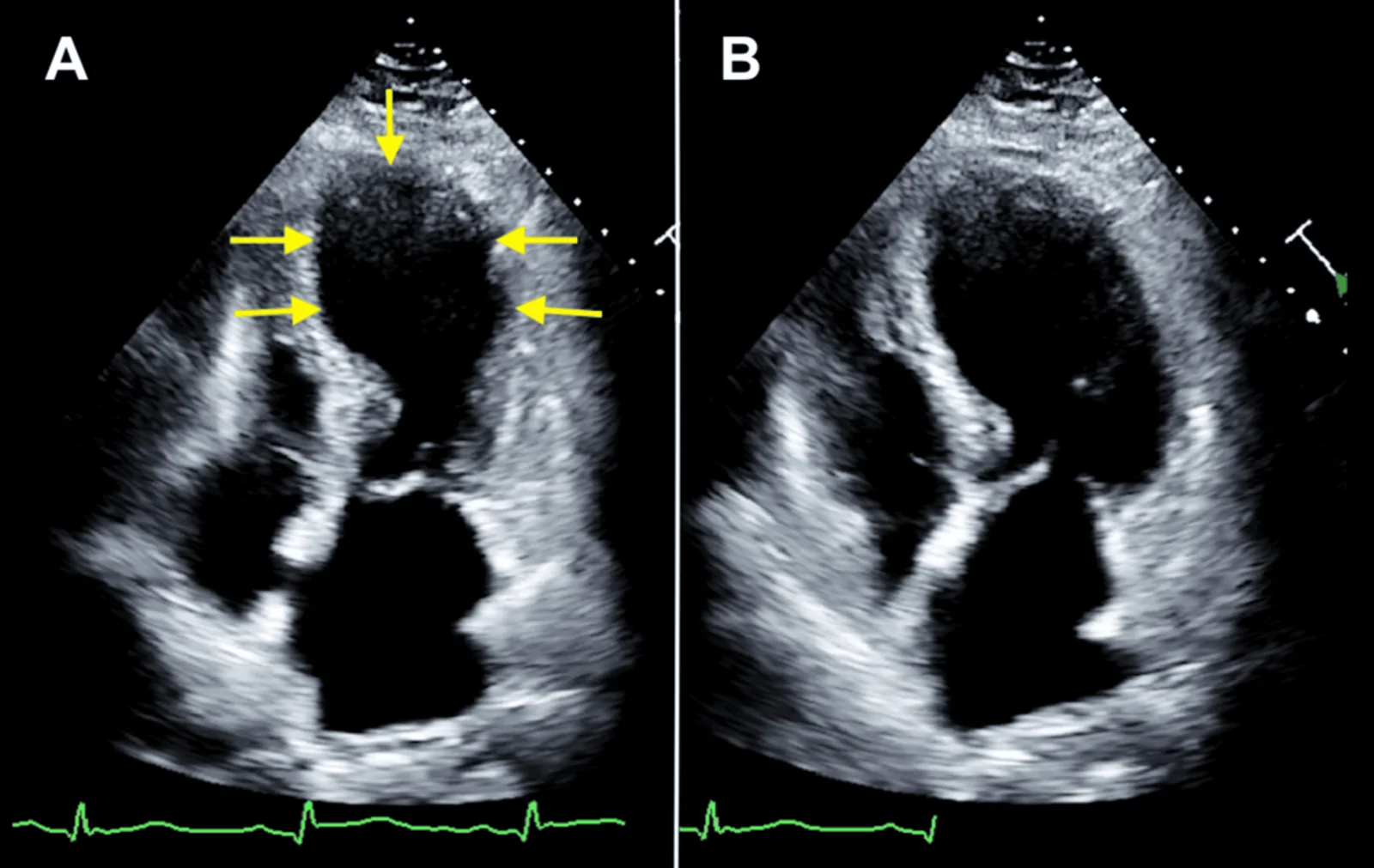
Day 1 echocardiogram showing systolic (A) and diastolic (B) apical four-chamber views Yellow arrows indicate akinetic left ventricular segments involving the mid anterior, mid inferoseptal, mid inferior, mid anterolateral, apical anterior, apical septal, apical inferior, apical lateral walls, and apex. Left ventricular ejection fraction was 35%

During hospitalization, she was monitored continuously for arrhythmias and treated with guideline-directed medical therapy. Her dyspnea gradually resolved over several days. Repeat echocardiography on Day 5 demonstrated a modest improvement in LVEF to 39%, although persistent apical akinesis remained evident (Figures 2A, 2B). She was discharged with outpatient cardiology follow-up.

**Figure 2:**
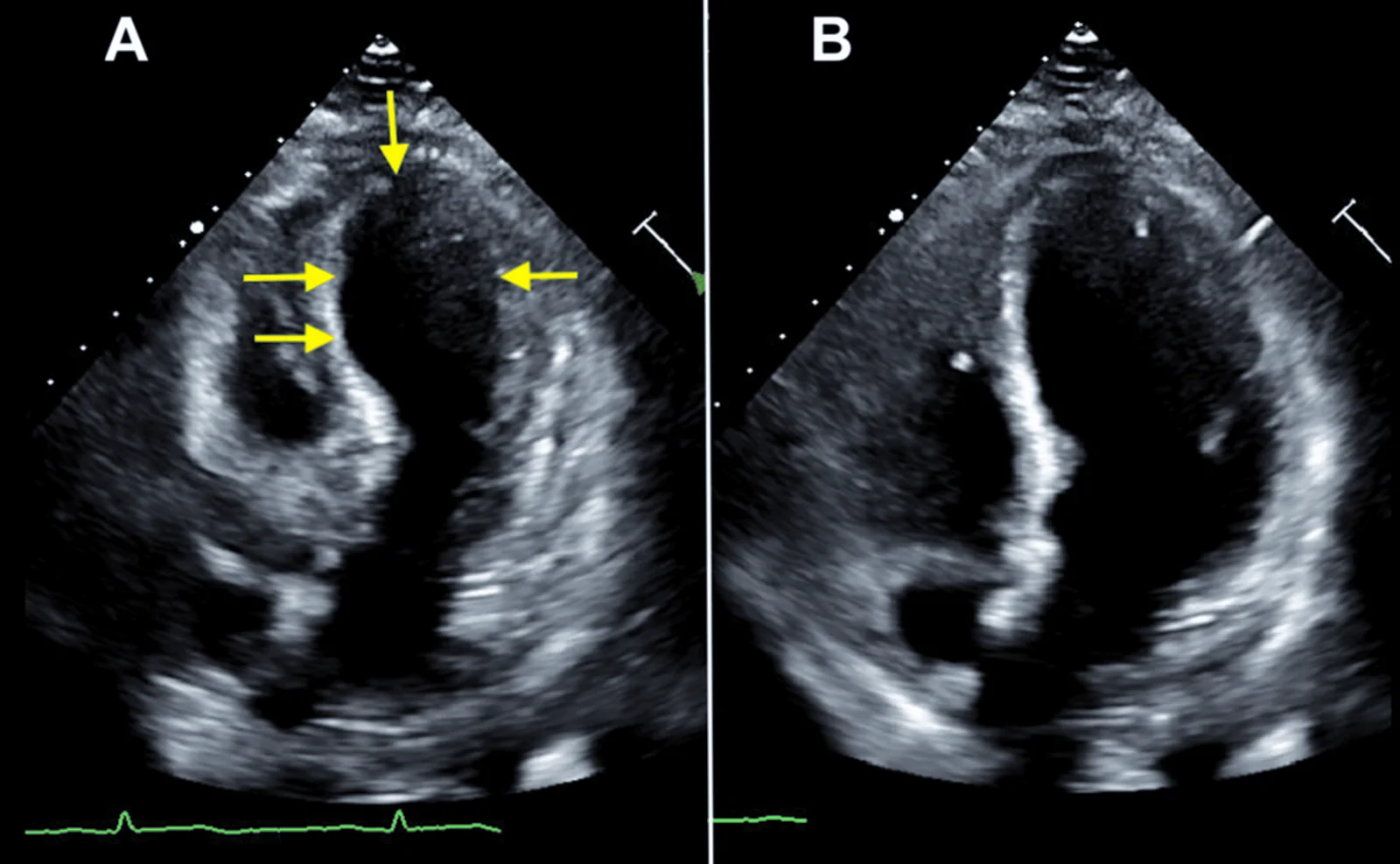
Day 5 echocardiogram showing systolic (A) and diastolic (B) apical four-chamber views Yellow arrows indicate persistent akinesis of the apical left ventricular segments with partial recovery of left ventricular systolic function. Left ventricular ejection fraction improved to 39%

At outpatient follow-up on Day 50, repeat echocardiography demonstrated persistent apical akinesis with no meaningful improvement in LV systolic function. The LVEF remained essentially unchanged at 38% (Figures 3A, 3B).

**Figure 3:**
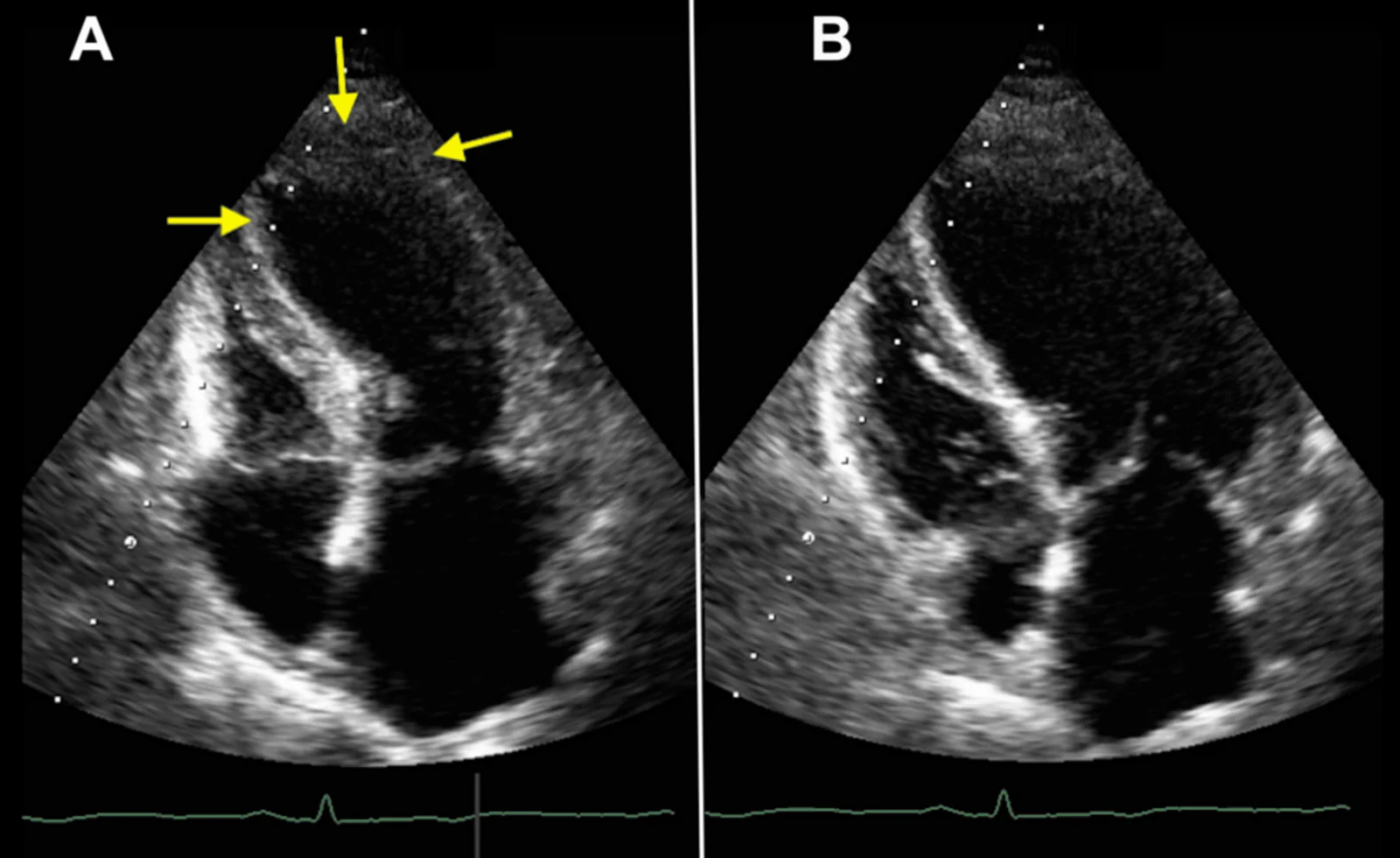
Day 50 echocardiogram showing systolic (A) and diastolic (B) apical four-chamber views Yellow arrows indicate persistent akinesis of the apical left ventricular segments. The left ventricular ejection fraction remained at 38%

She underwent elective hiatal hernia repair on Day 83 without perioperative cardiac complications. The procedure resulted in complete resolution of her chronic left shoulder discomfort. At four-month follow-up (Day 126), she had returned to baseline functional status, including a regular walking program and daily activities. Repeat echocardiography continued to demonstrate persistent apical akinesis with only minimal improvement in LV systolic function (LVEF 40%) despite complete clinical recovery (Figures 4A, 4B). Serial clinical, biochemical, and echocardiographic findings during follow-up are summarized in Table 1.

**Figure 4:**
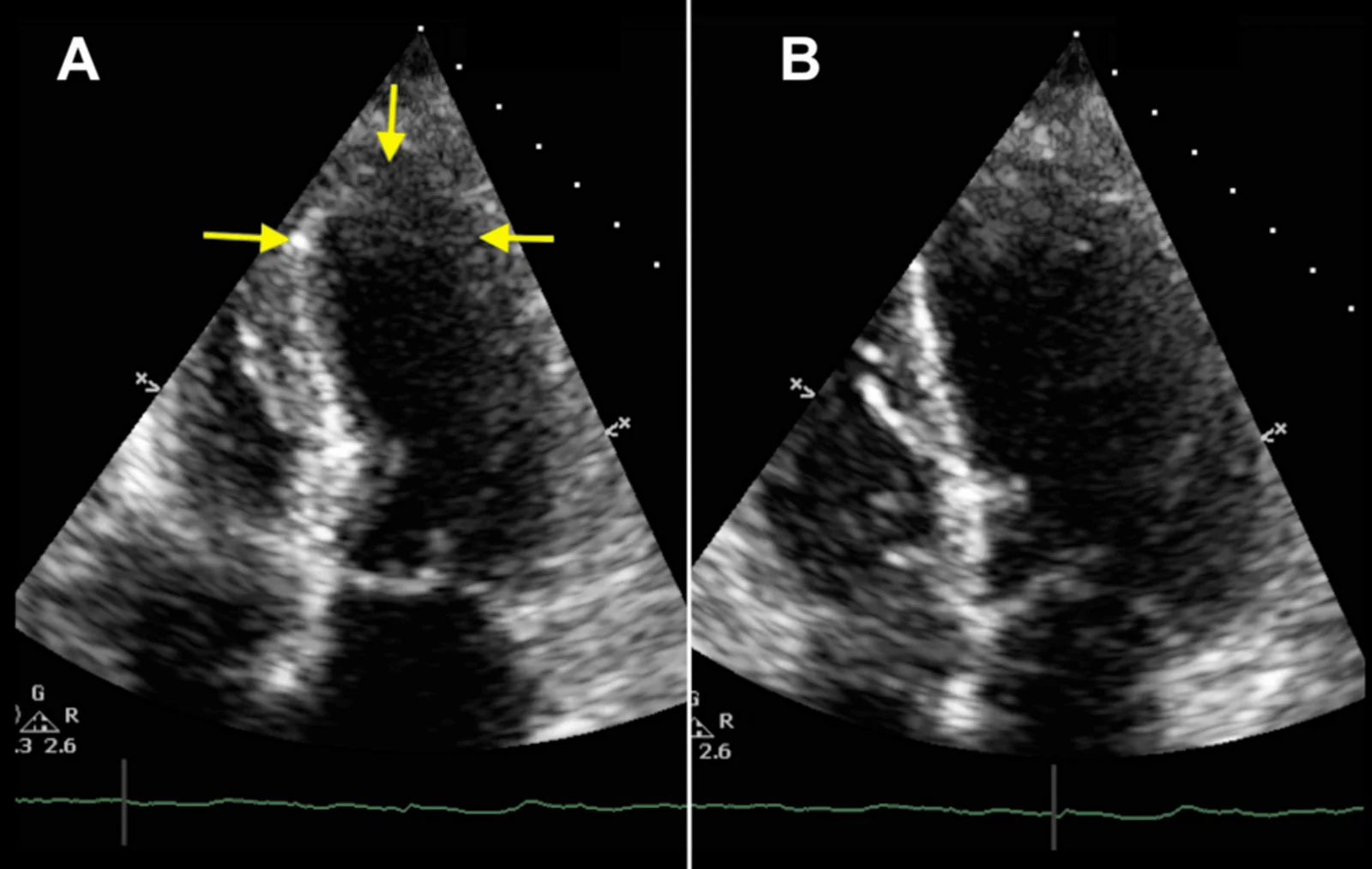
Day 126 echocardiogram showing systolic (A) and diastolic (B) apical four-chamber views Yellow arrows indicate persistent akinesis of the apical left ventricular segments. Left ventricular ejection fraction was 40%

**Table 1:** Serial clinical, biochemical, and echocardiographic findings during follow-up LVEF was calculated using Simpson's biplane method LVEF: left ventricular ejection fraction; LAD: left anterior descending; hs-troponin: high-sensitivity troponin

Parameter	Day 1	Day 5	Day 50	Day 126
Clinical status	Nausea, vomiting, epigastric discomfort, and dyspnea	No chest pain, no dyspnea, and not in acute distress	Asymptomatic; functional status improving	Baseline functional status and regular walking program
hs-troponin (ng/L)	9,848 (peak)	Not performed	Not performed	Not performed
LVEF (%)	35	39	38	40
Regional wall motion	Akinesis of mid anterior, mid inferoseptal, mid inferior, mid anterolateral, apical anterior, apical septal, apical inferior, and apical lateral segments and apex; hyperkinesis of all basal segments	Akinesis of apical anterior, apical septal, apical inferior, and apical lateral segments and apex; hypokinesis of mid anterior, mid anteroseptal, and mid inferoseptal segments; basal segments normal	Persistent akinesis of the apical segments with hypokinesis of the mid septal segments; remaining segments normal	Persistent akinesis of the apical segments with hypokinesis of the mid septal segments; remaining segments normal
Coronary angiography	Mild nonobstructive disease; approximately 20% LAD narrowing beyond the first diagonal	Not performed	Not performed	Not performed

## Discussion

TTS is generally considered a reversible cardiomyopathy, with previous studies demonstrating substantially faster recovery of LV function in most patients with TTS. Lee, in a serial echocardiographic study of 48 patients, reported improvement in mean LVEF to 49% within one week, with normalization to 58% ± 6.8% by six weeks [11]. By comparison, our patient's LVEF was 39% at Day 5 and 38% at Day 50, remaining approximately 20 percentage points below the mean value reported at six weeks in that cohort.

However, increasing evidence suggests that recovery may be incomplete in some patients despite apparent clinical improvement. Scally et al. reported that a year after TTS, many patients remained symptomatic with reduced exercise capacity and subclinical cardiac dysfunction despite normalized LVEF and biomarkers [12]. Similarly, the REgistry of TAKOtsubo Syndrome registry study by Almendro-Delia et al. (n = 1,463) demonstrated that delayed LV recovery (>10 days) was associated with increased mortality. Patients with delayed recovery had a median recovery time of 20 days (Q1-Q3: 12-35 days) [13]. In contrast, our patient had not recovered at 126 days, approximately six times the median recovery time and more than three times the upper quartile (35 days) reported for the late recovery group in that registry, supporting the growing recognition that recovery after TTS may be heterogeneous. While these serial echocardiographic findings are compatible with delayed recovery from TTS, cardiac magnetic resonance imaging was not performed, and the available data therefore cannot establish TTS as definitively responsible for the persistent regional abnormality.

The mechanisms underlying delayed recovery remain incompletely understood. Cardiac magnetic resonance studies have demonstrated myocardial edema during the acute phase of TTS, with persistent edema reported up to four months after presentation [14]. In addition, evidence of ongoing low-grade myocardial inflammation has been observed more than 12 months after the acute event [12]. These findings raise the possibility that persistent myocardial inflammation could contribute to prolonged ventricular dysfunction. However, we did not perform tissue characterization or inflammatory assessment in our patient, so this remains a hypothetical explanation rather than one demonstrated by the present case.

Another notable feature of this case was the marked elevation in hs-troponin levels. Troponin elevation in TTS is typically modest compared with ACS. Couch et al. in their meta-analysis demonstrated significantly lower troponin levels in TTS than ACS, with an absolute difference of approximately 75 times the upper limit of normal [3]. Nevertheless, occasional reports have documented substantially higher troponin values in TTS. One published case described a troponin I concentration exceeding 24,000 ng/L [15]; however, LV systolic function normalized during early follow-up. In contrast, our patient demonstrated persistent ventricular dysfunction despite a peak hs-troponin level of 9,848 ng/L.

Incidentally, a large hiatal hernia was identified during the initial evaluation. The patient also reported chronic left shoulder discomfort predating presentation that resolved following hernia repair. Similar coexistence of TTS and giant hiatal hernia has been reported previously [16].

This report has several limitations. Cardiac magnetic resonance imaging was not performed; therefore, preexisting myocardial scar, alternative cardiomyopathies, myocarditis, or residual myocardial injury could not be definitively excluded. Coronary narrowing was assessed by visual estimation, with angiography showing noncritical stenosis and normal flow that did not meet criteria for invasive assessment with intravascular ultrasound for more precise evaluation. In the absence of intracoronary imaging and cardiac magnetic resonance imaging, myocardial infarction with nonobstructive coronary arteries related to the mildly narrowed, hazy LAD segment could not be definitively excluded. Serial hs-troponin measurements beyond the peak value were not available, limiting assessment of the biomarker release pattern. In addition, the duration of follow-up was insufficient to determine whether eventual normalization of regional wall motion abnormalities and LV systolic function would occur.

## Conclusions

This case highlights an atypical recovery pattern in TTS, characterized by persistent apical akinesis and only minimal improvement in LV systolic function more than four months after the acute event despite complete clinical recovery. These findings suggest that recovery of myocardial function may not always parallel symptomatic improvement.

The marked elevation in hs-troponin, together with persistent ventricular dysfunction despite the absence of obstructive coronary artery disease, illustrates the variable clinical spectrum of TTS. This case also emphasizes the importance of maintaining a high index of suspicion for TTS in patients presenting with features suggestive of ACS, even when concurrent noncardiac conditions may complicate the clinical presentation.

Overall, this report adds to the growing recognition that TTS may have a more heterogeneous recovery pattern than traditionally appreciated. Further studies are needed to identify predictors of delayed ventricular recovery, clarify the mechanisms underlying persistent myocardial dysfunction, and define the role of follow-up imaging in patients with delayed functional recovery.
